# Shenqi Fuzheng Injection Reverses Cisplatin Resistance through Mitofusin-2-Mediated Cell Cycle Arrest and Apoptosis in A549/DDP Cells

**DOI:** 10.1155/2018/8258246

**Published:** 2018-10-16

**Authors:** Ying Xiong, QiuYu Zhao, LiYan Gu, ChunYing Liu, Chun Wang

**Affiliations:** ^1^Basic Medical College, Liaoning University of Traditional Chinese Medicine, Shenyang, China; ^2^Minimally Invasive Diagnosis and Treatment Section, The Fourth Affiliated Hospital of Liaoning University of Traditional Chinese Medicine, Shenyang, China; ^3^Key Laboratory of Ministry of Education for TCM Viscera-State Theory and Applications, Ministry of Education of China (Province-Ministry Co-Construct), Liaoning University of Traditional Chinese Medicine, Shenyang, China

## Abstract

The goal of this evaluation was to examine the mechanisms of Shenqi Fuzheng injection (SFI), an extract made from the plants* Radix Astragali *and* Radix Codonopsis*, in the process of chemotherapy sensitivity in non-small-cell lung cancer (NSCLC) cells. We investigated the expression of mitofusin-2 (Mfn2), a mitochondrial GTPase that may be related to chemoresistance, and found that Mfn2 expression was lower in human cisplatin-resistant lung carcinoma A549/DDP cells than in cisplatin-susceptible A549 cells. Chemosensitivity to cisplatin was restored in A549/DDP cells following supplementation in conjunction with SFI treatment, the effect of which we evaluated* via* cell cycle, apoptosis, and cell signaling analysis. We found that the combined use of A549/DDP cells with SFI and cisplatin enhanced cell cycle arrested in the G_2_/M phase, which was accompanied by upregulation of p53 and p21 protein expression and induced mitochondrial apoptosis in conjunction with the upregulation of Bax and the downregulation of Bcl-2 protein expression. Moreover, cell cycle arrest and mitochondrial apoptosis coincided with the upregulation of Mfn2 expression, which, in turn, was related to the increased mitochondrial membrane permeabilization and elevated reactive oxygen species. In summary, our findings suggest that the effect of SFI in increasing chemotherapy sensitivity in cisplatin resistance of NSCLCs occurs through cell cycle arrest and the initiation of mitochondrial apoptosis involved in the upregulation of Mfn2 expression.

## 1. Introduction

Lung cancer is one of the most frequently diagnosed cancers across the globe, with non-small-cell lung cancer (NSCLC) accounting for nearly 85% of all lung cancer diagnoses [1]. Cisplatin-based chemotherapy is one of the most efficient therapeutic treatments for NSCLC; however, acquired drug resistance that develops during treatment is now a large barrier that negatively impacts the survival rate of patients [2]. Therefore, investigation of the molecular mechanisms of cisplatin resistance and the identification of effective strategies that promote cisplatin sensitivity will greatly improve the efficacy of NSCLC therapeutics.

Prior evaluations have indicated that numerous mechanisms may prompt cisplatin resistance, among which the evasion of apoptosis and inappropriate cell proliferation greatly account for instances of drug resistance [3, 4]. Mitochondrial GTPase mitofusin-2 (Mfn2) gene is a protein that remains in the mitochondrial outer membrane and plays a pivotal part in mitochondrial fusion, thereby managing mitochondrial morphology and activities [5]. Aside from its main participation in mitochondrial fusion, the dysfunction of Mfn2 has been suggested in various critical roles including in controlling cell proliferation, apoptosis, and autophagy [6, 7]. Previous research has shown that the expression of Mfn2 is reduced in tumor tissue versus in adjacent nontumorous tissues and that it negatively corresponds with tumor size and tumor prognosis [8, 9]. Interestingly, cell proliferation, apoptosis, and autophagy are usually associated with cisplatin resistance in NSCLC [3, 4, 10]. Nevertheless, our understanding is that the potential role that Mfn2 plays in NSCLC cisplatin resistance has not yet been identified.

**Figure 1:**
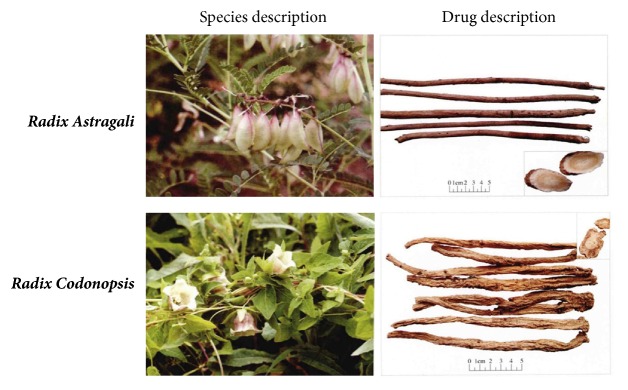
Species and drug description of SFI.

In China, with the goal of enhancing chemosensitivity and the therapeutic impact of cisplatin-based chemotherapy, numerous traditional Chinese medicinal herbs have been broadly combined with cisplatin-based chemotherapy for NSCLC. One such medicinal herb option is the Shenqi Fuzheng injection (SFI), which is developed from an extract of* Radix Astragali *and* Radix Codonopsis *mixed at a ratio of 1:1 and prepared as an injectable treatment (Figure 1).* Radix Astragali, *the dried root of* Astragalus membranaceus* (Fisch.) Bge. var.* mongholicus* (Bge.) Hsiao, has been used as a therapeutic for overall weakness; ongoing illnesses; and spleen deficiency syndromes including anorexia, fatigue, and diarrhea. In addition,* Radix Astragali* has been documented to have immunomodulatory, antioxidant, anti-inflammatory, and antitumor effects [11–13].* Radix Codonopsis, *the dried root of* Codonopsis pilosula* (Franch.) Nannf.,* Codonopsis pilosula *Nannf. var.* modesta* (Nannf) L. T. Shen, has been used for the treatment of lethargy, poor appetite, thirst, indigestion, chronic diarrhea, archoptoma, chronic anemia, and leukemia [14]. SFI was approved in 1999 by the State Food and Drug Administration of the People's Republic of China as an antitumor treatment [15, 16]. Consequently, there have been many trials published on the combination of SFI and either cisplatin or other chemotherapeutic drugs for NSCLC, gastric cancer, breast cancer, and other malignant tumors [17–20]. These trials have demonstrated the efficacy of a SFI–systematic chemotherapy combination in sensitizing tumors and lowering the toxicity of standard chemotherapy. Nevertheless, whether or not SFI is a chemoresistance reversal agent and what the underlying mechanisms of SFI in increasing chemotherapy sensitivity are still unknown.

In the present study, we investigated whether SFI could reverse chemoresistance in the cisplatin-resistant lung carcinoma A549/DDP cell line and also evaluated the mechanism(s) underlying the antitumor effects in the induction of cell cycle arrest and apoptosis.

## 2. Materials and Methods

### 2.1. Preparation of SFI

**Figure 2:**
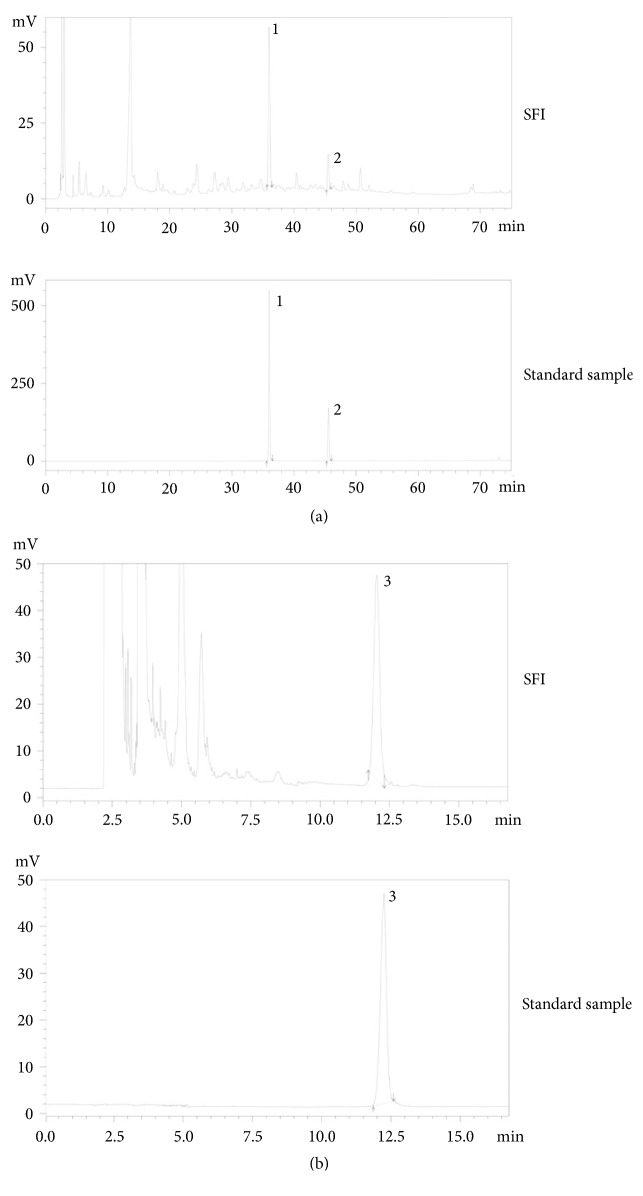
**HPLC data of SFI.** (a) and (b) Ultraviolet scatter diagram and evaporative light scattering diagram (upper panel) and standard sample (lower panel). The peaks indicate the presence of calycosin-7-O-*β*-glucoside (1), lobetyolin (2), and astragaloside IV (3), which confirms the authenticity of SFI.

SFI (Z19990065) came from Livzon Pharmaceutics Ltd. (Zhuhai, China). SFI is an injectable compound that is prepared from two types of Chinese medicinal herbs (*Radix Astragali *and* Radix Codonopsis*) in a ratio of 1:1. Calycosin-7-O-*β*-glucoside, lobetyolin, and astragaloside IV are the main compounds of* Radix Astragali* and* Radix Codonopsis* and thus are ideal markers for SFI [15]. The composition of SFI was confirmed by high performance liquid chromatography (HPLC) (Figure 2).

### 2.2. Cell Culture

The A549 human lung adenocarcinoma cell line (cisplatin-susceptible cells) and the A549/DDP variant cell line (cisplatin-resistant cells) were acquired from the Cancer Hospital at the Chinese Academy of Medical Sciences (Beijing, China) and cultured in Roswell Park Memorial Institute (RPMI) 1640 media with 10% fetal bovine serum (HyClone Laboratories, Logan, UT, USA) and 1% penicillin and streptomycin (Gibco, Carlsbad, CA, USA) in a humid atmosphere of 5% CO_2_/95% air. To retain the drug-resistant phenotype, 2 *μ*g/mL of cisplatin was supplemented in the A549/DDP media [21].

### 2.3. Cell Chemoresistance Capacity and Cell Viability Assay

A Cell Counting Kit-8 (CCK-8) (Vazyme, Nanjing, China) was used to examine the cell chemoresistance capacity and cell viability, as our previous investigation detailed [22]. Briefly, A549 or A549/DDP cells (5 × 10^3^ cells/well) were seeded in 96-well microplates and incubated overnight, and then treated with different concentrations of cisplatin (i.e., 0, 31.25, 62.5, 125, 250, and 500 *μ*g/mL). After incubation for more than 24 hours, cell viability was assayed* via* CCK-8 and the cell chemoresistance capacity was evaluated by the resistance index (RI), according to the following formula: half maximal inhibitory concentration (IC_50_) of A549/DDP cells ÷ IC_50_ of A549 cells.

The cell viability of SFI or cotreatment of cisplatin with SFI in A549/DDP cells was also determined by counting viable cells with CCK-8 assay. As for the treatment with SFI alone, the cells were incubated with different concentrations of SFI (i.e., 0, 0.4, 0.8, 1.6, 3.2, 6.4, 12.8, 25.6, and 51.2 mg/mL) for 26 hours. Furthermore, for the cotreatment of cisplatin with SFI, the cells were pretreated with IC_5_, IC_10_, and IC_20_ of SFI (2, 3.78, and 35.18 mg/mL) for 2 hours; subsequently, 40 *μ*g/mL of cisplatin (or about the concentration of IC_20_) was added for another 24 hours of incubation prior to assay for cell viability. The absorbance was measured at 460 nm.

### 2.4. Drug Combination Studies

We performed a combination index (CI), utilizing data acquired from the CCK-8 assay to examine the synergism among cisplatin and SFI on the prevention of cell growth in the A549/DDP cells. The A549/DDP cells were treated with a continuous ratio that was established with drug IC_50_ values determined using Chou and Talalay's method [23]. Three independent experiments were performed separately. CI values < 1 indicated synergism; CI values = 1 indicated an additive effect; and CI values > 1 indicated antagonism. The data were processed with the CalcuSyn software (Biosoft, Oxford, UK).

### 2.5. Immunocytochemical Assay

The A549 and A549/DDP cells developed on glass coverslips received low concentrations of cisplatin (18.3 *μ*g/mL, or about the concentration of IC_10_) for 24 hours or 48 hours. After treatment, the cells were secured in 2% formaldehyde for 10 minutes at room temperature. Then, the Mfn2 protein expressed in the cells was detected by Mfn2 antibody (1:200). The MetaMorph/DP10/BX41 image analyzing system (UIC/Olympus, US/JP) was used to analyze the results.

### 2.6. Western Blot Analysis

Western blot analyses were conducted according to protocols in a previous publication [24]. Following treatment, A549 or A549/DDP cells were lysed and the proteins were extracted, quantified, and then moved onto a polyvinylidene fluoride (PVDF) membrane. Immunoblots were analyzed with antibodies against Mfn2 (Abcam, Cambridge, UK); p53 (CST, Boston, MA, USA); p21 (CST, Boston, MA, USA); cleaved caspase-3 (SAB, Pearland, TX, USA); cleaved polyadenosine diphosphate-ribose polymerase (PARP; SAB, Pearland, TX, USA); Bax (CST, Boston, MA, USA); Bcl-2 (CST, Boston, MA, USA); and *β*-actin (SAB, Pearland, TX, USA). The proteins were then viewed with an ECL kit (Thermo Fisher Scientific, Waltham, MA, USA).

### 2.7. Flow Cytometry-Based Cell Cycle and Apoptosis Assay

A549/DDP cells were seeded in 6-well plates and pretreated by SFI (2, 3.78, and 35.18 mg/mL) for 2 hours, then incubated with cisplatin for another 24 hours. Cells were harvested for cell cycle distribution analysis by propidium iodide (PI) staining* via* flow cytometry and the results were analyzed by ModFit 3.0 (BD Accuri™ C6: BD Biosciences, San Jose, CA, USA). As for the apoptosis assay, cells were fluorescence stained by Annexin V-fluorescein isothiocyanate (FITC) apoptosis detection kit (SAB, Pearland, TX, USA) [25]. The percentages of apoptotic cells were determined by use of a flow cytometer and analyzed by the Cell Quest Pro software (Becton, Dickinson and Company, Franklin Lakes, NJ, USA) [22]. To confirm the mechanisms of cell cycle arrest and apoptosis-induced by coincubation with cisplatin and SFI, cells were pretreated with the reactive oxygen species (ROS) scavenger N-acetyl-cysteine (NAC) (2.5 mM) for 3 hours. Then, SFI and cisplatin were similarly processed as discussed above.

### 2.8. Hoechst Staining

Following coincubation with cisplatin and SFI, A549/DDP cells were secured in 70% ethanol and then incubated along with 10 *μ*g/mL bisbenzimide trihydrochloride (Hoechst 33258) staining solution (Sigma-Aldrich, St. Louis, MO, USA) for 30 minutes. After washing with phosphate-buffered saline (PBS), the cells were viewed under a fluorescence microscope (Leica, Wetzlar, Germany).

### 2.9. Measurement of Mitochondrial Membrane Potential by JC-1 Dye

Depolarization of mitochondrial membrane potential is characteristic of apoptosis, and the mitochondrial membrane potential (MMP) was quantified using the JC-1 dye (Invitrogen, Carlsbad, CA, USA) based on the details of a previous publication [26]. Following treatment with cisplatin and SFI, cells were dyed with JC-1 and analyzed with a FACS Calibur flow cytometer (BD Biosciences, San Jose, CA, USA). JC-1 is present as a monomer at lower concentrations with green fluorescence and gathers in the mitochondria, presenting with orange-red fluorescence as it aggregates. In healthy cells, JC-1 was aggregated into mitochondria depending on the polarized MMP, while, in apoptosis cells, JC-1 was released from the mitochondria for the depolarized MMP and existed as a monomer in the cytoplasm. Throughout apoptosis, there is an interruption in the mitochondrial membrane potential that can be observed by way of the change in fluorescence from red to green.

### 2.10. Measurement of ROS Production

Intracellular oxidative stress ROS assay kit (GENMED, Shanghai, China) was used to evaluate ROS production in A549/DDP cells after cisplatin and SFI treatment. Cells were stained by use of the dichlorodihydrofluorescein diacetate (DCFH-DA) method and visualized under a fluorescence microscope at the excitation wavelength of 540 nm and an emission wavelength of 590 nm (Olympus, Tokyo, Japan). Nonfluorescent DCFH-DA is cell-permeable and oxidized in the presence of ROS to form dihydroethidium (DHE), which determines the levels of ROS in the intracellular. ROS was determined by comparing the changes in fluorescence intensity with the control cells. Alternatively, the ROS content was measured by FACSCalibur flow cytometer (Becton, Dickinson and Company, Franklin Lakes, NJ, USA) and analyzed on Cell Quest software (BD FACS™ Calibur Flow Cytometer Cell Quest Software; BD Biosciences, San Jose, CA, USA). ROS was determined by comparing the changes in fluorescence intensity with those of the control [27].

### 2.11. Statistical Analysis

Every experiment was performed a minimum of 3 times. The data are expressed in the format of mean ± standard deviation. A statistical comparison among various groups was performed with analysis of variance using the Statistical Package for the Social Sciences version 15.0 software (IBM Corp., Armonk, NY, USA). A* P* value of < 0.05 indicated significance, while NS denotes no significant difference (*P* > 0.05).

## 3. Results

### 3.1. Downregulation of Mfn2 Is Part of the Chemoresistance of A549/DDP

To determine the chemoresistance ability of A549/DDP cells treated with cisplatin, we calculated the IC_50_ of cisplatin-susceptible A549 cells and cisplatin-resistant A549/DDP cells. The RI value was 8.89, demonstrating their cisplatin resistance capacity.

**Figure 3:**
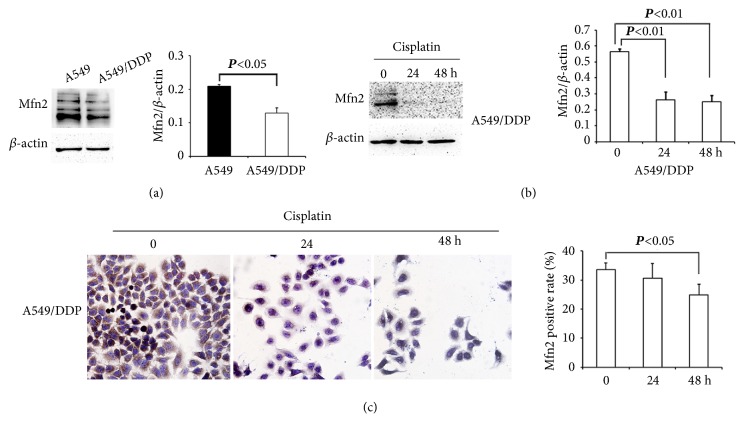
**Downregulation of Mfn2 with increased cisplatin resistance in A549/DDP cells.** (a) The expression level of Mfn2 in A549 and A549/DDP cells was measured by Western blot analysis and quantified by densitometry. Mean ± standard deviation, n = 3. (b and c) A549/DDP cells were cultured for 0, 24, and 48 hours in the absence or presence of cisplatin (40 *μ*g/mL). The expression level of Mfn2 was measured by Western blot and immunocytochemical assays. A demiquantization value by densitometry is shown on the right.

As a tumor suppressor gene,* Mfn2* plays large roles in managing cell proliferation and apoptosis [6, 7]. To analyze whether the Mfn2 protein was associated with chemoresistance, we compared the Mfn2 expression level in cisplatin-susceptible A549 and cisplatin-resistant A549/DDP cells. The outcomes revealed that cisplatin-susceptible A549 cells had a greater level of Mfn2 expression, while cisplatin-resistant A549/DDP cells had a lower expression than that of the A549 cells (Figure 3(a)). Next, we exposed the A549/DDP cells to a low concentration of cisplatin (18.3 *μ*g/mL, or about the concentration of IC_10_) for 24 hours and 48 hours and observed that Mfn2 level was significantly reduced with time, as assessed by Western blotting and immunohistochemistry (IHC) assays (Figures 3(b) and 3(c)).

### 3.2. SFI Reverses the Chemoresistance of A549/DDP Cells to Cisplatin

**Figure 4:**
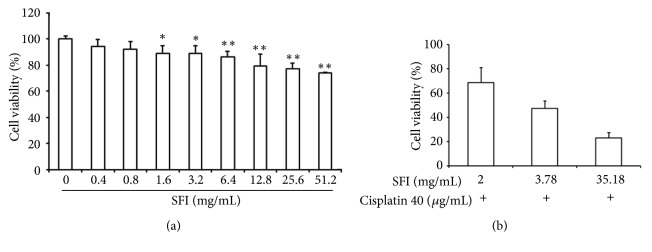
**SFI reverses the cisplatin resistance of A549/DDP cells.** (a) Direct cytotoxic effect of SFI on A549/DDP cells. A549/DDP cells were treated with various concentrations (0, 0.4, 0.8, 1.6, 3.2, 6.4, 12.8, 25.6, and 51.2 mg/mL) of SFI for 26 hours. The IC_5_, IC_10_, and IC_20_ values were 2, 3.78, and 35.18 mg/mL, respectively. Each data point represents the mean ± standard deviation of results from four individual measurements (*∗*:* P *< 0.05; *∗∗*:* P* < 0.01). (b) Cocytotoxic effect of cisplatin and SFI on A549/DDP cells. A549/DDP cells were pretreated with IC_5_, IC_10_, and IC_20_ of SFI (2, 3.78, and 35.18 mg/mL) for 2 hours and then 40 *μ*g/mL of cisplatin (about the concentration of IC_20_) was added for another 24 hours. The cell viabilities were 68.9%, 47.4%, and 23.2%, respectively.

To examine the potential role of SFI in the reversal of chemoresistance to cisplatin, we initially evaluated the cytotoxic impact of SFI on A549/DDP cells. SFI prevented the abundant generation of A549/DDP cells in a dose-dependent fashion, and the IC_5_, IC_10_, and IC_20_ values were 2, 3.78, and 35.18 mg/mL, respectively (Figure 4(a)).

We used Chou and Talalay's method (1984) [23] on MATLAB (MathWorks, Natick, MA, USA) to analyze the interaction (i.e., synergistic, additive, or antagonistic) of the combination of cisplatin and SFI on the A549/DDP cell lines. The calculated CI_cisplatinIC50+SFIIC20_ value for the A549/DDP cell lines was 0.79. Thus, the triple combination of cisplatin and SFI was harmonious in A549/DDP cells* via* isobologram analysis and representative curves.

Next, we pretreated A549/DDP cells with IC_5_, IC_10_, and IC_20_ (2, 3.78, and 35.18 mg/mL) of SFI for 2 hours and then 40 *μ*g/mL cisplatin (about the concentration of IC_20_) was added for another 24 hours of incubation before cell viability assay. The outcomes revealed that the combined treatments of cisplatin with SFI ended up with the cell viabilities of 68.9%, 47.4%, and 23.2%, respectively (Figure 4(b)). In addition, cotreatment of 40 *μ*g/mL of cisplatin and 3.78 mg/mL of SFI (IC_10_) significantly inhibited A549/DDP cell growth, as indicated by the nearly 50% reduction observed in cell viability rate.

### 3.3. Combination of Cisplatin and SFI Halts Cell Cycle in the G_2_/M Phase

**Figure 5:**
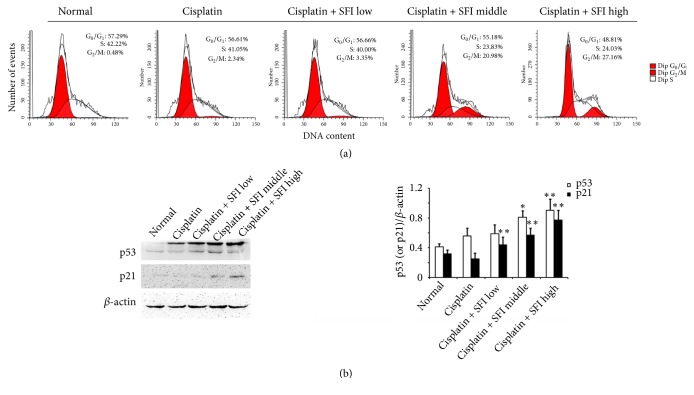
**Cotreatment with cisplatin and SFI induces cell cycle arrest in A549/DDP cells.** A549/DDP cells were pretreated with various concentrations (2, 3.78, and 35.18 mg/mL) of SFI for 2 hours and then exposed to cisplatin (40 *μ*g/mL) for another 24 hours. (a) Cell cycle distribution by PI staining and DNA contents were determined by flow cytometry. (b) Cell lysates were prepared and subjected to immunoblotting with antibodies to p53, p21, and *β*-actin. Data are presented in the format of mean ± standard deviation of three independent experiments (*∗*:* P *< 0.05, versus the cisplatin group; *∗∗*:* P *< 0.01, versus the cisplatin group).

We analyzed the dose-dependent alterations in the proportion of cells in different phases of the cell cycle following the combined cisplatin + SFI treatment to establish whether the growth inhibitory impact of SFI was induced by a certain deviation of cell cycle progression or not. A549/DDP cells were pretreated with the IC_5_, IC_10_, and IC_20_ of SFI for 2 hours and then exposed to cisplatin (40 *μ*g/mL) for another 24 hours. As shown in Figure 5(a), treatment with cisplatin and various concentrations of SFI led to cell cycle arrest, as reflected by an accumulation of the G_2_/M phase cells. We further observed that the combination of cisplatin and SFI treatment significantly prompted additional cells to go into the G_2_/M phases, and the percentage of G_2_/M phases in the cisplatin + SFI high group was ~56-fold larger than in the normal group, while the cisplatin group was only 4.8-fold larger than the normal group (the percentages of G_2_/M phase cells: 0.48% in the normal group; 2.34% in the cisplatin group; and 27.16% in the cisplatin + SFI high group). In addition, cisplatin and SFI cotreatment induced a reduction in cell numbers in the G_0_/G_1_ phase, but the decreased proportion was not statistically different upon contrasting with that in the cisplatin-only treatment group (*P *> 0.05).

As is well known, p53 is prompted by DNA damage checkpoint kinases to concurrently manage the G_1_/S and G_2_/M cell cycle checkpoints* via *the transcriptional induction of the cyclin-dependent kinase inhibitor p21, the downstream signaling molecule of p53 [28]. Moreover, in cisplatin-sensitive cells, cisplatin raises the p53-protein level and the expression of p21; conversely, in cisplatin-resistant cells, the expressions of p53 and p21 decrease following cisplatin exposure [22]. Therefore, we examined p53 and p21 expression in A549/DDP cells upon cisplatin and SFI treatment. A low expression of p53 was noted in cisplatin-resistant A549/DDP cells, while such was persistently increased following exposure to cisplatin (40 *μ*g/mL) alone and during cotreatment with SFI. In contrast to the normal group, a notable increase was seen in the cisplatin + SFI medium and high groups (Figure 5(b)). Moreover, cisplatin and SFI cotreatment effectively upregulated p21 expression (Figure 5(b)).

### 3.4. Combination of Cisplatin and SFI Prompts Apoptosis in A549/DDP Cells

**Figure 6:**
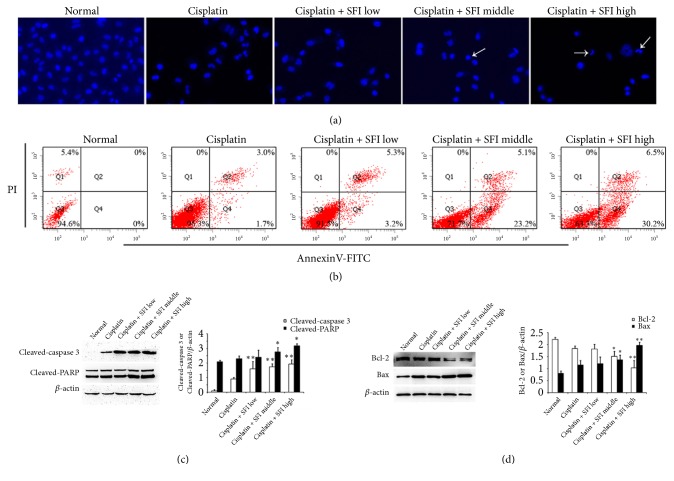
**Cotreatment with cisplatin and SFI induces cell apoptosis in A549/DDP cells.** A549/DDP cells were pretreated with various concentrations (2, 3.78, and 35.18 mg/mL) of SFI for 2 hours and then exposed to cisplatin (40 *μ*g/mL) for another 24 hours. (a) Apoptosis determined by Hoechst staining. “→” shows apoptosis cells with nuclear condensation. (b) Apoptosis determined by Annexin V-FITC/PI staining. Each cytogram consists of data showing live cells (PI- and FITC-negative) in Q3; early apoptotic population (FITC-positive) in Q4; mid- to late-stage apoptosis (PI- and FITC-positive) in Q2; and necrotic/end-stage apoptotic cells (PI-positive and FITC-negative) in Q1. (c) Cell lysates were prepared and subjected to immunoblotting with antibodies to cleaved caspase 3, cleaved-PARP, Bcl-2, Bax, and *β*-actin. Data are presented in the format of mean ± standard deviation of three independent experiments (*∗*:* P *< 0.05, versus the cisplatin group; *∗∗*:* P *< 0.01, versus the cisplatin group).

Next, we explored the apoptotic mechanisms that may restore the sensitivity to cisplatin. A549/DDP cells were treated with a combination of cisplatin and SFI and subsequently subjected to Hoechst staining and then flow cytometry analysis. As can be seen in Figure 6(a), cisplatin and SFI cotreatment induced significant apoptosis with nuclear condensation as observed by fluorescence microscope. Meanwhile, the apoptosis was further confirmed by double staining with Annexin V-FITC and PI. The cisplatin and SFI cotreatment increased the overall percentage of apoptotic cells (Q2 quadrant + Q4 quadrant) from 4.7% (cisplatin group) to 8.7% (cisplatin + SFI low group), 28.3% (cisplatin + SFI medium group), and 36.7% (cisplatin + SFI high group) [Figure 6(b)]. To confirm that the combination of cisplatin and SFI induced apoptosis, the initiation of caspase-3 typical for prompting apoptosis and the deactivation of PARP, a DNA repair factor, were analyzed by immunoblotting. Caspase-3 initiation and PARP deactivation/cleavage were gradually upregulated following SFI cotreatment with cisplatin [Figure 6(c)]. Next, we viewed the protein expression of the proapoptotic protein Bax and the antiapoptotic protein Bcl-2 by Western blotting.  Figure 6(d) shows cotreatment with cisplatin and SFI significantly raised the protein levels of Bax and lowered the protein expressions of Bcl-2, respectively.

### 3.5. Combination of Cisplatin and SFI Upregulates Mfn2 Protein Expression

**Figure 7:**
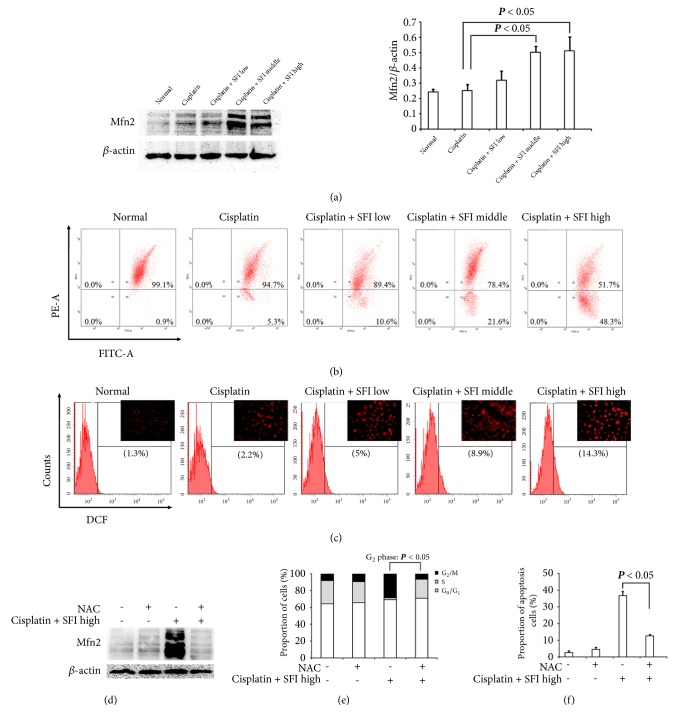
**Mfn2 is involved in reversing cell cycle inhibition and cell apoptosis upon cotreatment with cisplatin and SFI.** (a, b, and c) A549/DDP cells were pretreated with various concentrations (2, 3.78, and 35.18 mg/mL) of SFI for 2 hours and then exposed to cisplatin (40 *μ*g/mL) for another 24 hours. After drug intervention, (a) cell lysates were prepared and subjected to immunoblotting with antibodies to Mfn2 and *β*-actin; (b) cells were incubated with JC-1 and analyzed by flow cytometry; and (c) cells were labeled with DCFH-DA and the fluorescence intensity of the oxidized product DCF in individual cells was detected by flow cytometry and fluorescence microscopy. (d, e, and f) A549/DDP cells were pretreated with or without 2.5 mM of NAC, followed by cisplatin (40 *μ*g/mL) and SFI (35.18 mg/mL) cotreatment. After drug intervention, (d) cell lysates were prepared and subjected to immunoblotting with antibodies to Mfn2 and *β*-actin; (e) cell cycle distribution by PI staining and DNA contents were determined by flow cytometry; and (f) apoptosis was determined by Annexin V-FITC/PI staining and analyzed by flow cytometry.

We further investigated Mfn2 protein expression by Western blotting in cisplatin-resistant A549/DDP cells prompted by the cotreatment of cisplatin and various concentrations of SFI. As shown in Figure 7(a), we observed a gradual increase in Mfn2 protein expression with cisplatin and SFI cotreatment in A549/DDP cells. Furthermore, we investigated MMP and ROS, the two hallmarks related to mitochondrial function. Gradually decreased MMP and elevated ROS concurrent with an Mfn2 protein expression increase were observed, as demonstrated in Figures 7(b) and 7(c). However, upon the pretreatment of A549/DDP cells with the ROS scavenger NAC (2.5 mM) followed by cisplatin and SFI exposure, we found that NAC indeed downregulated Mfn2 protein expression [Figure 7(d)], reversed cell cycle inhibition [Figure 7(e)], and diminished cell apoptosis [Figure 7(f)], which suggested a role of ROS in cisplatin and SFI cotreatment-mediated upregulation of Mfn2 in cisplatin-resistant A549/DDP cells.

## 4. Discussion

The gene encoding Mfn2, which has been mapped to chromosome 1q36.22, is expressed in different types of tissues, such as the brain, heart, and skeletal muscle [29]. Aside from its main participation in mitochondrial fusion, mitochondrial morphology, and mitochondrial function [30], dysfunction of this Mfn2 is also linked with an assortment of pathological conditions, such as Charcot–Marie–Tooth disease type 2A, diabetes mellitus type 2, obesity, and cancer [8, 9, 29, 31, 32]. Besides, as a tumor suppressor gene,* Mfn2* additionally plays large parts in managing cell proliferation and apoptosis [6, 7]. In the current evaluation, we first compared the IC_50_ of A549/DDP cells with that of A549 cells and found that the RI was 8.89. This result indicated that A549/DDP cells had substantial cisplatin resistance. Subsequently, we found that the expression of Mfn2 protein was downregulated in cisplatin-resistant A549/DDP cells when contrasted with the cisplatin-susceptible A549 cells, and a continued decrease occurred in A549/DDP cells when exposed to low concentrations of cisplatin. These findings suggested that Mfn2 might be a potential molecular target that takes part in the mechanisms of cisplatin resistance in lung cancer cells.

In clinical use, SFI has antitumor synergy impacts in connection with chemotherapy [17–20]. Our results highlighted the direct cytotoxic effect of SFI on A549/DDP cells, which is in line with the findings of some previous reports on the anticancer properties of certain biologically active compounds, such as saponins, flavonoids, and echinocystic acid, that have been isolated from* Radix Astragali* and* Radix Codonopsis*, which inhibit tumor growth via the induction of apoptosis [11, 33]. In addition, cotreatment of cisplatin with SFI was superior to treatment with cisplatin alone for the inhibition of A549/DDP cell growth. Our outcomes confirmed that SFI can undo the cisplatin resistance of A549/DDP cells, similarly to the case of other traditional Chinese herbal medicines that have been previously found to reverse tumor multidrug resistance [34].

Cell cycle retardation is an important antiproliferation mechanism in cancer. Cotreatment of A549/DDP cells with cisplatin and different concentrations of SFI significantly culminated in cell cycle arrest in the G_2_/M phase, which suggested that modulation of the cell cycle might have a causative part in the reversal of cisplatin resistance effects of SFI in NSCLC cells. Human* p53* is described as the “guardian of the genome” since it is able to manage the expression of some genes and microRNAs that impact cellular processes, such as proliferation, DNA repair, apoptosis, and autophagy [35]. Though the protein level and transcriptional activities of p53 are maintained at low levels in some cisplatin-resistant tumor cells [36, 37], DNA damage regulated by cisplatin causes an increase in p53 protein stability and consequently impacts cell fate via the raised transcription of p21, which then obstructs cyclin-dependent kinases 1 and 2 (CDK-1 and -2) and causes G_1_/S and G_2_/M cell cycle arrest [38]. In our study, cisplatin and SFI cotreatment induced G_2_/M cell cycle arrest in cisplatin-resistant A549/DDP cells, after which point, a rise in p53 and p21 protein expression occurred, which suggested that SFI reversed the cisplatin resistance of A549/DDP via the upregulation of p53 and the protein expression of its downstream signaling molecule p21 contributing to preferential cell growth inhibition, respectively.

Apoptosis is activated and takes place via two primary pathways: intrinsic and extrinsic. The Bcl-2 family of proteins contributes to the intrinsic pathway, also known as the mitochondrial pathway, by managing mitochondrial penetrability and the liberation of cytochrome C. The antiapoptotic proteins Bcl-2 and Bcl-xL remain in the outer mitochondrial wall and prevent cytochrome C liberation, while the proapoptotic Bcl-2 proteins Bax and Bad remain in the cytosol but move to the mitochondria after activation of death signaling, at which time they advance the liberation of cytochrome C. Released cytochrome C can directly induce the cleavage of caspases, which then activate caspase-9 and caspase-3 [39]. Our data demonstrated that the apoptosis of cisplatin-resistant A549/DDP cells prompted by cisplatin in combination with SFI was mediated via the Bcl-2 family of proteins, indicating that the intrinsic pathway regulated by mitochondria could have a pivotal part in apoptosis triggered by way of the combination of the two drugs. Therefore, triggering of the mitochondria and the induction of mitochondrial apoptosis may partly explain the role of SFI in restoring cisplatin sensitivity in NSCLC cells.

Previous investigations have shown that Mfn2 overexpression results in a cell cycle arrest at the G_2_/M phase and induces caspase-independent apoptosis in colorectal cancer cells [40]. Additionally, Mfn2 was determined to be a new immediate p53 target that could also have an apoptotic impact through Bax signaling and possibly prevent proliferation in HCC cells [6, 41, 42]. Besides, Mfn2 induces apoptosis through the Bax/Bcl-2 pathway and then activates the mitochondrial apoptotic pathway [6]. Our study indicated that cisplatin and SFI cotreatment suppressed the proliferation of cisplatin-resistant A549/DDP cells, halted the cell cycle in G_2_/M phages by increasing p53 and p21 protein expression, and induced cell apoptosis via the Bcl-2 family of proteins, which were all involved in the upregulation of Mfn2.

Mitochondria are powerful organelles that continuously experience fusion and fission of their outer and inner membranes. The balance between these processes ensures mitochondrial morphology and normal function [43]. Mitochondrial fusion enables the exchange of contents, DNA, and metabolites among nearby organelles, advancing their survival [44]. Mitochondrial fission is required for the precise mitochondrial movement and regulation of apoptosis via the separation of the most severely damaged mitochondria [45]. Mfn2 is essential for mitochondrial morphology and function, depending on its effects of promoting mitochondrial fusion and inhibiting mitochondrial fission. Moreover, Mfn2 can also reduce mitochondrial membrane potential and promote intracellular ROS. In contrast, ROS may further increase mitochondrial membrane permeability, resulting in mitochondrial apoptosis [46, 47]. In our work, we determined that cotreatment with cisplatin and SFI increased mitochondrial membrane permeabilization (characterized by decreased MMP) and elevated ROS levels, which are involved with the upregulation of Mfn2 in cisplatin-resistant A549/DDP cells. Therefore, we speculate that cotreatment with cisplatin and SFI upregulated Mfn2 protein expression in cisplatin-resistant NSCLC cells, which induced mitochondrial outer membrane permeabilization, translocation of Bax from the cytoplasm to the mitochondria, and initiation of cytochrome C release. Collectively, these outcomes suggested that SFI increased the cytotoxicity of cisplatin through the mitochondrial (intrinsic) apoptotic pathway in cisplatin-resistant A549/DDP cells.

## 5. Conclusion

In conclusion, to our knowledge, this is the first report that proposes the mechanism of SFI in restoring cisplatin sensitivity. Our results highlight the concept that cotreatment of cisplatin and SFI prompted cell cycle arrest and mediated cell apoptosis events that were part of the upregulation of Mfn2, suggesting the role of Mfn2 in chemoresistance in cancer cells. Furthermore, the rising Mfn2 protein expression in A549/DDP cells was related to the increased mitochondrial membrane permeabilization and elevated ROS. Conclusively, this study has revealed the mechanism of SFI in increasing chemotherapy sensitivity, which may be extremely important for its clinical pharmacology evaluation and its use as a chemoresistance reversal agent that can be further employed in clinical cancer treatment.

## Data Availability

The data used to support the findings of this study are available from the corresponding author upon request.
